# High order Fano resonances and giant magnetic fields in dielectric microspheres

**DOI:** 10.1038/s41598-019-56783-3

**Published:** 2019-12-30

**Authors:** Zengbo Wang, Boris Luk’yanchuk, Liyang Yue, Bing Yan, James Monks, Rakesh Dhama, Oleg V. Minin, Igor V. Minin, Sumei Huang, Andrey A. Fedyanin

**Affiliations:** 1 School of Electronic Engineering, Bangor University, Dean Street, Bangor Gwynedd, LL57 1UT UK; 2 Division of Physics and Applied Physics, School of Physical and Mathematical Sciences, Nanyang Technological University, Singapore, 637371 Singapore; 3 Faculty of Physics, Lomonosov Moscow State University, Moscow, 119991 Russia; 4 National Research Tomsk State University, Lenin Ave., 36, Tomsk, 634050 Russia; 5 National Research Tomsk Polytechnic University, Lenin Ave., 30, Tomsk, 634050 Russia; 6 Engineering Research center for Nanophotonics & Advanced Instrument, Ministry of Education, School of Physics and material Science, East China Normal University, Shanghai, 200062 PR China

**Keywords:** Optical physics, Optical materials and structures, Microresonators, Optics and photonics

## Abstract

We show that weakly dissipating dielectric spheres made of materials such as glass, quartz, etc. can support high order Fano resonances associated with internal Mie modes. These resonances, happening for specific values of the size parameter, yield field-intensity enhancement factors on the order of 10^4^–10^7^, which can be directly obtained from analytical calculations. Associated to these “super-resonances”, we analyze the emergence of magnetic nanojets with giant magnetic fields, which might be attractive for many photonic applications.

## Introduction

Fano resonances, resulting from the interference of broad and narrow excitation modes, have emerged as an interesting topic of research with promising applications in physical, chemical, and biological sciences^1,2^. The quality factor (sharpness), as well as the amplitude of Fano resonances increase for higher order modes. As an example, interference of a broad dipole (Rayleigh-like) mode and a narrow octupole mode provides a sharper resonance than that stemming from the interference between the dipole and quadrupole modes in plasmonic particles (see e.g. Fig. 3 in ref. ^1^). Therefore, excitation of these, higher-order Fano resonances^3–6^ might be beneficial to enhance the sensitivity of resonant nanostructures. An example of such high order Fano resonances (quadrupole, octupolar, hexadecapolar, and triakontadipolar) are those generated in optimized disk-ring silver plasmonic nanostructure^6^. Further progress towards higher order resonances in plasmonic nanostructures, however, has been limited by the large dissipation associated with metals, particularly in the visible range. On the contrary, dielectric materials might be the perfect platform to observe such effects, as the dissipation effects can be very small. In the following, we show that this is the case, and that realization of high order Fano resonances is possible in simple systems such as spherical particles, including both high index (e.g. n = 4) and moderate (e.g. n = 1.5) index materials.

**Figure 1:**
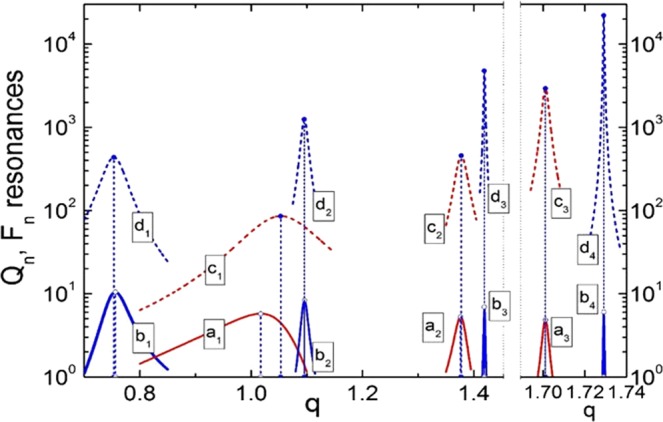
Partial scattering efficiencies ${Q}_{\ell }$ and ${F}_{\ell }$, related to scattering amplitudes, ${a}_{\ell }$ and ${b}_{\ell }$, and internal field amplitudes, ${c}_{\ell }$ and ${d}_{\ell }$, for a spherical particle with refractive i*n*dex *n* = 4 as a function of its size parameter *q*. Solid lines represent the partial scattering efficiencies ${Q}_{\ell }$, and dotted lines the internal partial efficiencies ${F}_{\ell }$. Electric amplitudes are shown in red color and magnetic amplitudes in blue. Note the change in the x-axis scale, introduced to present the sharp resonances found for $\ell \ge 3$.

**Figure 2:**
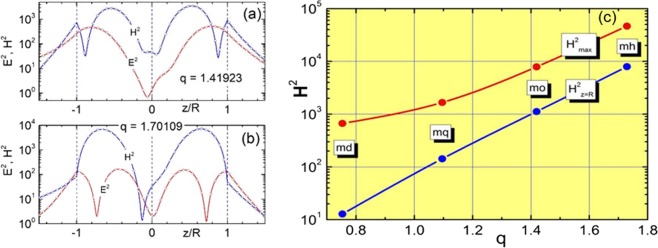
Electric and magnetic field distributions for a particle with refractive index n = 4 and size parameter (**a**) *q* = 1.41923 (magnetic octupole resonance) and (**b**) *q* = 1.70109 (electric octupole resonance). (**c**) Maximal values of H^2^ (normalized to the incidence ones) inside the particle (red dots) for the first four internal magnetic resonances. The intensity of the magnetic field at the particle surface, in the point opposite to the wave incidence, are also shown (blue dots). Smooth solid lines are shown just for better visibility. Particle refractive index *n* = 4.

To study the emergence of high-order resonances in spherical particles, we make use of the well-known solution of the scattering problem from a homogeneous sphere, i.e., the Mie theory. A brief summary of the main expressions allowing to compute the scattering (${Q}_{\ell }$) and internal (${F}_{\ell }$) fields associated to the different electric and magnetic multipolar modes supported by the particle ($\ell$ being the order) are given in the Methods section^7–9^. In Fig. 1 we present the characteristic positions of different resonances and maximal values of the corresponding scattering and internal field amplitudes ${Q}_{\ell }$ and ${F}_{\ell }$ for a spherical particle with refractive index *n* = 4. For modes above octupolar order $(\ell \ge 3)$ resonances are very sharp, and we show them in the right panel of Fig. 1 with higher resolution of the size parameter *q*. The observed fast increase in the amplitude of the internal resonances is accompanied by a huge growth of the electric and magnetic fields, as shown in Fig. 2. In particular, Fig. 2a,b display the intensity of electric and magnetic fields inside the particle at the electric and magnetic octupolar resonances, respectively. As can be seen, the enhancement of the magnetic field intensity can reach values that are up to two orders of magnitude larger than those of the electric field. For completeness, Fig. 2c shows the comparison between the maximal values of the magnetic field intensity inside the particle and at its surface, for the first four resonances supported. In the following, we extend this intensity enhancement effect to higher order resonances using moderate refractive index materials that are known to have very low dissipative losses, such as glass.

## High Order Fano Resonances

**Figure 3:**
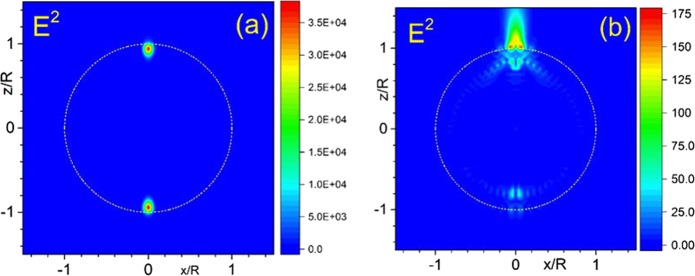
Distribution of intensity *E*^2^ in the $\{x,z\}$ plane, calculated by Mie theory, where all terms $\ell \le {\ell }_{\max }=41$ are taken into account in calculations (**a**) and the same distribution without the single $\ell =35$ resonant term (**b**).

The microscopic origin of the Fano resonance arises from the constructive and destructive interference of a narrow discrete resonance with a broad spectral line or continuum^1,2^. In our case, we are interested in studying Fano resonances arising from the interference between high order resonances and the broad spectrum provided by all the other modes. In Fig. 3, we illustrate this effect for the particle with refractive index *n* = 1.5 (non-absorbing, close the typical values of glasses) and size parameter *q* = 26.9419. These parameters correspond to a resonant magnetic mode excited inside the particle with partial wave order $\ell =35$. At the same time the total number of modes with a significant contribution for this size parameter in the Mie theory is ${\ell }_{{\rm{\max }}}=41$. In Fig. 3a, one can see the electric field intensity distribution in the {*x*,*z*} plane, where all modes with $\ell \le {\ell }_{{\rm{\max }}}$ are taken into account in calculations. In Fig. 3b we present the same picture where all $\ell$ terms are taken into account except the single resonant term with $\ell =35$. From the comparison one can immediately see that the single term $\ell =35$ produces around a 200 times increase of the intensity.

**Figure 4:**
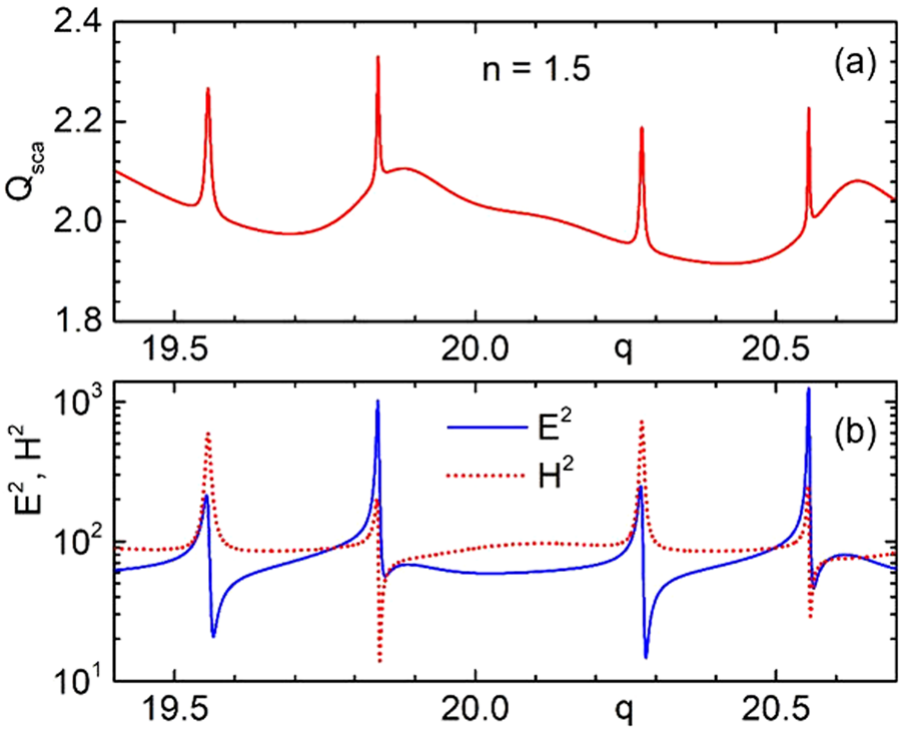
Scattering efficiency ${Q}_{sca}(q)$ for particle with refractive index $n=1.5$ near size parameter $q\approx 20$ (**a**). Electric and magnetic intensities on the surface for the same particle (**b**).

Due to the interference of broad and narrow spectral lines, we obtain characteristic Fano line-shapes in the far field scattering as well as for the electric and magnetic intensity spectrums, as shown in Fig. 4 for some size parameter range near $q\approx 20$. The emergence of the Fano shape in the scattering efficiency was analyzed in Ref. ^10^. These extra narrow resonances produce about 20% variation of signal in the scattering efficiency, but one-two orders of magnitude in the electric and magnetic field intensities. In Fig. 4b, we show the intensities spectra on the surface of the particle, i.e. at $\{x=y=0,\,z=R\}$. The wave is taken as propagating in the positive z-axis. Note that the intensities inside the particle can be up to one order of magnitudes higher, as can be seen, e.g., in Fig. 3a. We coin these high order Fano resonances, for which field-intensity enhancement factors can reach values on the order of 10^4^–10^7^, as “*super resonances*”. Note that the typical range of size parameters necessary to obtain such resonances obviously depend on the refractive index. For the moderate values of highly transparent materials like glass, the range of interest lies within $q\approx 10\div30$. In order to obtain these effects in the visible range of the spectrum, one should thus consider particle sizes from a few to few-tens of micrometers.

**Figure 5:**
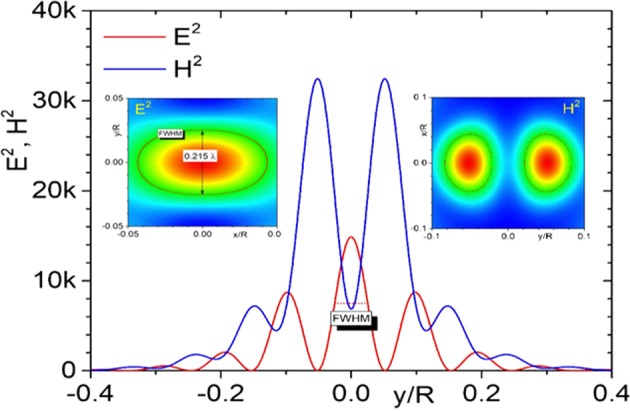
Distribution of the electric $${E}^{2}$$ and magnetic $${H}^{2}$$ fields along the *y*-axis at the output plane at $$z=R$$ for particle with refractive index $$n=1.5$$ and size parameter $$q=26.9419$$. It corresponds fields variations along the *y*-coordinate along the upper bright point in Fig. 3a. 2D field distributions within the $$\{x,y\}$$ planes are shown in the insets. The solid lines in the 2D insets indicate the position of the full width at half maximum (FWHM). One can see high light localization along the y-direction: for electric field the FWHM is $$0.215\,\lambda $$, less than the diffraction limit given by $$\,\lambda /2n=$$$$0.333\lambda .$$

Another important feature of high order Fano resonances is the high degree of field localization (beyond the diffraction limit) inside the particle and on the particle surface, as seen for example in Fig. 5. This is related to the formation of regions with high values of local wave vectors, an effect that has been discussed, e.g., in the frame of the theory of superoscillations^11–21^. According to this theory, the local wave vector can be understood as the local gradient of the phase ${{\bf{k}}}_{local}=\nabla \varPhi =\nabla {\bf{E}}/E$. High values of *k*_*local*_ can be created, e.g., by metamaterial lenses, by phase and amplitude masks and also in free space optics including vortices and knots. An optical vortex presents a singularity (zero intensity point) with circulating field phase around, given by a topological number *n* (total change of phase around singularity is 2*πn*). Taking a path with radius *r* around the singular point one can see that the change of 2*πn* in phase occurs along the length 2*πr*, i.e. the corresponding gradient of phase has an order *n*/*r*, which means that local wave vector tends to infinity at $r\to 0.$ This property also follows from the energy-time form of the Heisenberg uncertainty principle $\Delta E\cdot \Delta t\ge \hslash /2$. Using $E=N\hslash \omega$ and $t=\Phi /\omega$ this uncertainty can be reformulated in terms of number of photons and their phase $\Delta N\Delta \Phi \ge 1/2$. Differentiating this formula is easy to find that ${k}_{local}\propto \nabla N/{N}^{2}$, i.e. high local wave numbers can be reached in the vicinity of the singularity.

**Figure 6:**
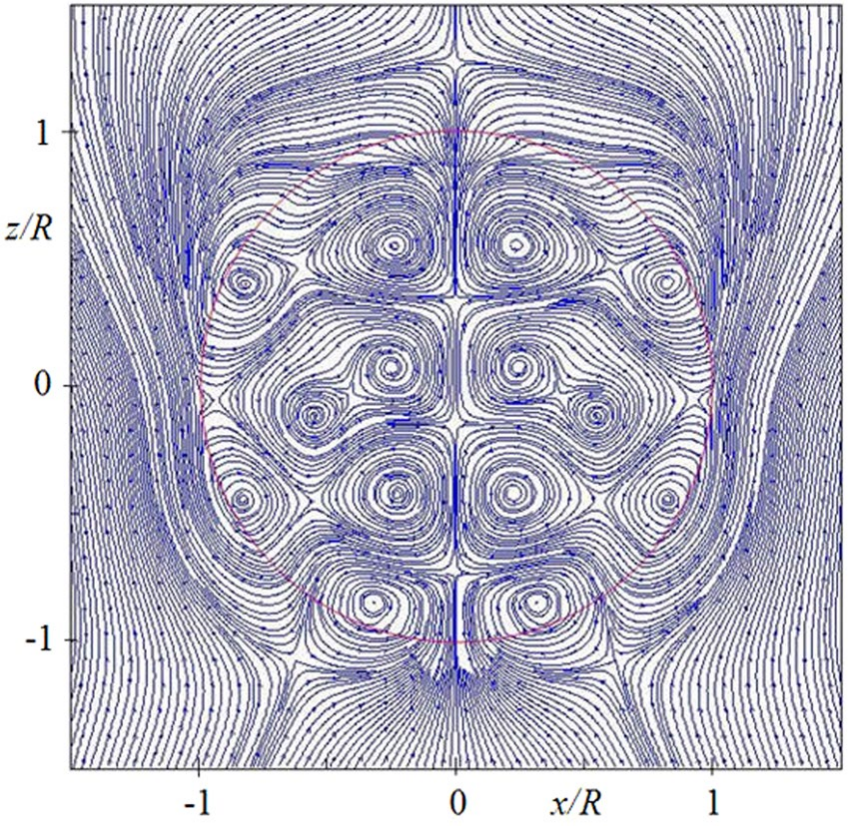
Poynting vector distribution for the particle with refractive index *n* = 4 and *q* = 2.

Optical nanovortices can be created around plasmonic and dielectric nanoparticles^22–29^. In dielectric particles optical vortices arise when size parameter exceeds some value, which depends on its refractive index^30,31^. These vortices can be easily seen in the plot of Poynting’s vector, as seen, for example, in Fig. 6. It corresponds to a distribution of displacement currents in {*x*,*z*} plane. Circular currents create magnetic fields according to the Biot-Savart law, which states that the magnetic field at center of a current loop is given by $B=\mu I/2R$. For a current *I* = 1 A and a loop with radius *R* = 10 nm one can create magnetic induction *B* = 63.8 Tesla (T)

In Fig. 6 one can see vortices with size $\lambda /10\pi$. Loops of displacement current are of the order of 20 nm in visible range. It shows the ability to create high magnetic fields above 10 T even with small silicon particles. High enhancement of localized electric and magnetic fields with small silicon nanodimer was experimentally demonstrated in Ref. ^32^. Calculation results show the enhancement of magnetic field was twice larger than the electric field enhancement^32^. Note that magnetic fields of light are quite small. For example, 1 µJ, 100 fs laser pulse focused to a 200 µm^2^ area produces 5 × 10^16^ W/cm^2^ or electric fields of the order of 10^7^ V/cm, as reported in Ref. ^33^. This yields magnetic induction in vacuum of just about 3 T. However, inside a glass particle, as shown above, magnetic field can be enhanced above $3\cdot {10}^{4}$ times (see in Fig. 5), which may produce magnetic induction values of the order of 10^5^ T for the same exciting laser (close to interatomic magnetic fields). With this field one can expect nonlinear dependence $\mu =\mu (H)$, i.e. magnetic nonlinear optics, where variations of the refractive index $n=\sqrt{\varepsilon \mu }$ is caused by purely magnetic effects. Such magnetic nonlinear optics can be realized if two conditions are fulfilled: 1) the dissipation is quite small and 2) the magnetic nonlinear response significantly exceeds the electric nonlinear response due to non-linearity $\varepsilon =\varepsilon (E)$.

**Figure 7:**
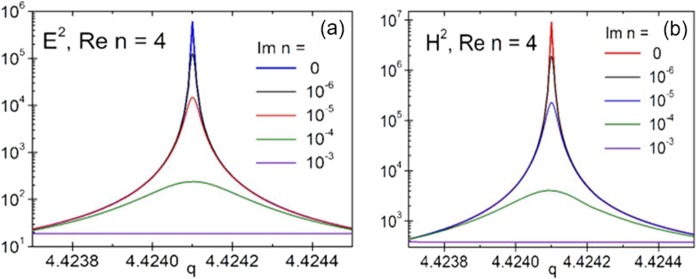
Effect of dissipation for the case of a magnetic super resonance excited in a particle with refractive index *n* = 4 and size parameter $q=4.4241$. Dissipation of the level of $\text{Im}n\approx {10}^{-5}$ yields two order of magnitude suppression in the maximal field values, while at $\text{Im}n\approx {10}^{-3}$ these resonances practically disappear.

It is not easy to fulfill these two conditions. First, super resonances are quite sensitive to dissipation. For example, in Fig. 7 we show the evolution of a magnetic super resonance for a particle with refractive index *n* = 4 and size parameter *q* = 4.4241 as a function of the imaginary part of the refractive index. The fields in the case of a purely non-dissipative material at this super resonance reach values around 10^6^ (for electric intensity *E*^2^) and 10^7^ (for magnetic intensity *H*^2^). However, with as low dissipation as ${\rm{Im}}n\le {10}^{-3}$ these resonances are strongly suppressed. Probably, the necessary level of dissipation to see super resonances in the optical range can be reached for materials with refractive index less than two (e.g. glass). While it is questionable whether it is possible to realize such high order resonances using high-index material (e.g. Si) in the optical range, it maybe be still possible to do so in the IR range, where the level of dissipation can be very low.

**Figure 8:**
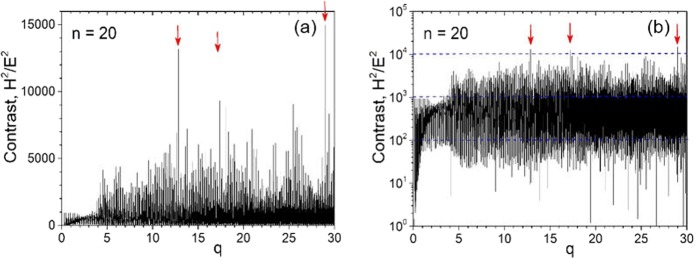
Contrast between magnetic and electric field enhancements for a particle with refractive index *n* = 20 as a function of the size parameter presented in decimal (**a**) and logarithmic (**b**) scales.

The second condition means that we need a high contrast of magnetic to electric field enhancement, i.e. ${H}^{2}/{E}^{2}\ge {10}^{4}$. Calculations show that this contrast is small (typically less than 10) for a particle with refractive index $n\le 2$. Also, it decreases with the increase of the size parameter. However, with high refractive index it is possible to provide high contrast at some particular values of *q*, as shown in Fig. 8. There are three values of *q* at which this can be achieved. Roughly speaking, for ${H}^{2}/{E}^{2} > {10}^{4}$ magnetic optical effects are dominant, for ${10}^{3}\le {H}^{2}/{E}^{2}\le {10}^{4}$ both, electric and magnetic contributions in nonlinear effects are comparable, and for ${H}^{2}/{E}^{2}\le {10}^{3}$ conventional nonlinear optics related to nonlinearity $\varepsilon =\varepsilon (E)$ is dominant. Big values of *n* for some ceramics^34^, e.g. $\varepsilon =180$ for ${{\rm{Ti}}}_{2}{{\rm{Nb}}}_{10}{{\rm{O}}}_{29}$ can be seen in the microwave region. However_,_ it is not clear how small dissipation can be reached. In any case, with super resonances in dielectrics, it might be possible to realize magnetization induced optical nonlinearity. Until now, nonlinear effects in magnetization-induced optical nonlinearity were observed mainly in thin ferromagnetic films^35,36^.

Beyond their possible application in novel non-linear phenomena, the ability to create highly localized fields with enhancement values on the level of 10^5^, both, inside the particle and outside in the near field region, open new venues in many modern applications, such as in photonic magnetic nanojet generation^31^, white light superlens nanoscopy^30^ and surface-enhanced Raman spectroscopy (SERS)^37^. Also, it might serve as a means for optimization of whispering gallery modes, widely used for telecommunication applications in, e.g., wavelength division multiplexing (WDM) schemes^38,39^.

## Conclusions

In conclusion, we reveal novel, super-resonance modes supported by dielectric spheres. These resonances, present for all multipolar orders, exhibit Fano line shapes and have extraordinarily high associated electric and magnetic field enhancements, which increase linearly with the multipolar order. The phenomenon can be observed at visible frequencies using simple glass microspheres, which may allow enhancing the magnetic field of light (which is typically small) by a few orders of magnitude. We believe these super-resonances are an attractive platform for some promising applications, like e.g. enhanced absorption effect, ablation caused by magnetic pressure, or those mentioned above.

## Methods

The Mie theory express the scattered fields in the form of superposition of partial waves in terms of spherical harmonics^7–9^. According the Mie theory the total scattering efficiency is presented by sum of partial scattering efficiencies:

1 $${Q}_{sca}=\mathop{\sum }\limits_{\ell =1}^{\infty }({Q}_{\ell }^{(e)}+{Q}_{\ell }^{(m)}),\,{Q}_{\ell }^{(e)}=\frac{2(2\ell +1)}{{q}^{2}}{|{a}_{\ell }|}^{2},\,{Q}_{\ell }^{(m)}=\frac{2(2\ell +1)}{{q}^{2}}{|{b}_{\ell }|}^{2}.$$

where each partial efficiency corresponds to the radiation of the $\ell$-th order multipole. Terms ${Q}_{\ell }^{(e)}$ and ${Q}_{\ell }^{(m)}$ describe the radiation related to the electric and magnetic polarizabilities, respectively. In the following, we will discuss transparent dielectrics with $\text{Im}\,\varepsilon =0$, so ${Q}_{ext}={Q}_{sca}$. The electric, ${a}_{\ell }$, and magnetic, ${b}_{\ell }$, scattering amplitudes for nonmagnetic materials with relative magnetic susceptibility $\mu =1$, and dielectric permittivity $\varepsilon ={n}^{2}$ (*n* being the refractive index of the particle material) are given by:

2 $${a}_{\ell }=\frac{{\Re }_{\ell }^{(a)}}{{\Re }_{\ell }^{(a)}+i\,{\Im }_{\ell }^{(a)}},\,{b}_{\ell }=\frac{{\Re }_{\ell }^{(b)}}{{\Re }_{\ell }^{(b)}+i\,{\Im }_{\ell }^{(b)}},$$

where ${\Re }_{\ell }$ and ${\Im }_{\ell }$ functions are defined as follows:

3 $${\Re }_{\ell }^{(a)}=n{\psi ^{\prime} }_{\ell }(q){\psi }_{\ell }(nq)-{\psi }_{\ell }\,(q){\psi ^{\prime} }_{\ell }(nq),\,{\Im }_{\ell }^{(a)}=n{\chi ^{\prime} }_{\ell }(q){\psi }_{\ell }(nq)-{\chi }_{\ell }(q){\psi ^{\prime} }_{\ell }(nq),$$

4 $${\Re }_{\ell }^{(b)}=n{\psi ^{\prime} }_{\ell }(nq){\psi }_{\ell }(q)-{\psi }_{\ell }(nq){\psi ^{\prime} }_{\ell }(q),\,{\Im }_{\ell }^{(b)}=n{\chi }_{\ell }(q){\psi ^{\prime} }_{\ell }(nq)-{\psi }_{\ell }(nq){\chi ^{\prime} }_{\ell }(q).$$

Here, ${\psi }_{\ell }(z)=\sqrt{\frac{\pi \,z}{2}}{J}_{\ell +\frac{1}{2}}(z)$, ${\chi }_{\ell }(z)=\sqrt{\frac{\pi \,z}{2}}{N}_{\ell +\frac{1}{2}}(z)$, where ${J}_{\ell +\frac{1}{2}}(z)$ and ${N}_{\ell +\frac{1}{2}}(z)$ are the Bessel and Neumann functions. The radius of the particle *R* enters in this theory through the dimensionless size parameter $q=\omega R/c=2\pi R/\lambda$, where $\omega$ is the angular frequency, *c* the speed of light, and $\lambda$ the radiation wavelength in vacuum. The prime in formulas (3), (4) indicates differentiation with respect to the argument of the function, i.e. ${\psi ^{\prime} }_{\ell }(z)\equiv d{\psi }_{\ell }(z)/dz$, etc. Electric and magnetic fields inside the particle are expressed through the internal scattering amplitudes given by

5 $${c}_{\ell }=\frac{in}{{\Re }_{\ell }^{(a)}+i\,{\Im }_{\ell }^{(a)}},\,{d}_{\ell }=\frac{in}{{\Re }_{\ell }^{(b)}+i\,{\Im }_{\ell }^{(b)}}.$$

Although the denominators of these amplitudes are the same as in amplitudes ${a}_{\ell }$ and ${b}_{\ell }$ in (2), which means that position of these resonances are close, the numerators of (5) never tends to zero. As a result the values of amplitudes ${|{c}_{\ell }|}^{2}$ and ${|{d}_{\ell }|}^{2}$ are not restricted by unity as amplitudes ${|{a}_{\ell }|}^{2}$ and ${|{b}_{\ell }|}^{2}$ in (2), but increase with values of size parameter and refractive index. To compare both type of resonances it is convenient to introduce partial internal scattering efficiencies, like in Eq. (1):

6 $${F}_{\ell }^{(e)}=\frac{2\,(2\ell +1)}{{q}^{2}}\,{|{c}_{\ell }|}^{2},\,{F}_{\ell }^{(m)}=\frac{2\,(2\ell +1)}{{q}^{2}}\,{|{d}_{\ell }|}^{2}.$$

Super-resonance modes are extremely sensitive to the size parameter *q*. Our tests reveal that a *q* sampling accuracy of 10^–4^ is required to ensure all modes were identified. This in turn leads to a supercomputing problem and parallelization of Mie code is required. We used a specially designed 10-parallel-thread Mie code for the computation, which takes about 4 hours to complete the calculation as in Fig. 8(a).
